# Extravasation of Intraarticular Fluid Injection Following Canine Elbow Arthroscopy: A Cadaveric Study

**DOI:** 10.1055/a-2648-6856

**Published:** 2025-07-16

**Authors:** Linus Rustemeyer, Matthias Galipaud, Peter Böttcher, Philipp A. Schmierer

**Affiliations:** 1 Small Animal Clinic Posthausen, Posthausen, Germany; 2 Swiss Data Science Center, Zürich, Switzerland; 3 Central German Competence Center for Small Animals, Leipzig, Germany

**Keywords:** elbow, arthroscopy, extravasation, dog, orthobiologics

## Abstract

**Objective:**

To investigate extravasation of contrast media injected intraarticularly, either immediately following elbow arthroscopy or after arthrocentesis using computed tomographic (CT) imaging at different time points.

**Study Design:**

Cadaveric dog model.

**Methods:**

A total of 16 elbows of eight canine cadavers (median age 10.2 years, 5.6 to 16.8 years; median weight 35.0 kg, 26.0 to 42.0 kg) were randomly assigned to the arthroscopy group with the contralateral elbow as control. Right after elbow arthroscopy, both elbows were injected with 2.5 mL contrast fluid. Elbow CT scans were obtained at seven time points. To detect a possible loss of contrast medium from the joint, both the total volume and intensity of the contrast fluid were measured intra- and extraarticularly using rendering software. To compare volume and intensity of contrast fluid, a simple linear model and a linear mixed effect model were used.

**Results:**

The total volume of contrast-enhanced fluid was increased (avg. difference: 5115 mm ^3^ ; linear model std. estimate: 1.69, std. error 0.10) and the total intensity decreased (avg. difference: 1330 Hounsfield Units; linear model std. estimate: −1.66, std. error 0.11) in the arthroscopy group compared with the control. Neither total volume nor total intensity of contrast-enhanced fluid changed significantly within 15 minutes.

**Conclusion:**

Extravasation of intraarticular injected contrast fluid after elbow arthroscopy without a significant effect of time could be shown. Injection of liquid therapeutics, e.g., orthobiologics, at a later point after arthroscopy should be considered.

## Introduction

Arthroscopy is one of the most frequently used diagnostic tools for elbow pathology in dogs. 1 2 3 Besides its significance as a diagnostic tool, arthroscopy also offers the opportunity of minimally invasive surgical treatment for many pathologic conditions of the elbow joint. 4 5

Agents like local anesthetics, 6 7 8 opioids, 6 9 glucocorticoids, 10 11 12 hyaluronic acid, 10 13 14 and also biological active agents such as platelet-rich plasma 10 13 15 16 and stem cells 14 17 are injected into affected joint(s). Although some surgeons perform joint injections immediately after the arthroscopic procedure, others prefer to inject at a later time point, because they speculate that, although arthroscopy is considered to be minimally invasive, the portals required for elbow arthroscopy might allow for leakage of the injected agent into the periarticular tissues, diminishing its intraarticular therapeutic potential.

In veterinary medicine Blumhagen and colleagues evaluated the effect of arthroscopy on extravasation scores after contrast fluid injection in a cadaveric dog model. 18 In their work they could not show a significant effect of an arthroscopy treatment on extravasation scores in 3D-CT models compared with an arthrocentesis control group. They also compared the effect of different volumes of injected contrast fluid and could show increased extravasation scores in the 4 mL versus the 2 mL group, regardless of arthroscopy treatment or arthrocentesis. 18

However, other veterinary and human studies have shown significant extravasation of irrigation fluid during arthroscopic procedures, making extravasation of intraarticular administered fluids likely. 19 20 21 22 The question also arises as to what effect a longer observation period could have on possible extravasation of intraarticular injected fluids. The purpose of this cadaveric study was to investigate extravasation of contrast media injected intraarticularly, either immediately following elbow arthroscopy or after simple arthrocentesis using computed tomographic (CT) imaging of the elbow region at different time points.

Our hypothesis was that there is a significantly increased extravasation of contrast media in the arthroscopy group compared with the arthrocentesis group, which increases gradually over the various time points.

## Materials and Methods

**Fig. 1:**
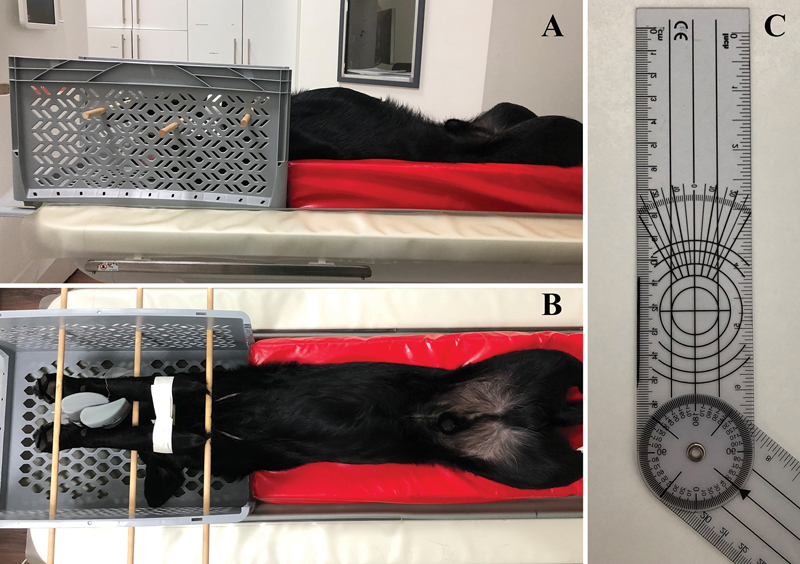
Experimental setup and specimen positioning. ( **A** ) Lateral and ( **B** ) ventro-dorsal views of a dog specimen in dorsal recumbency placed in a positioning mat with the front limbs fixed in a custom-made device with the elbow joints fixed at a 135-degree angle. ( **C** ) A goniometer was used to place the elbow joints in a physiologic standing angle of 135 degrees.

Dogs with body weight >20 kg, humanely euthanized in accordance with current international guidelines for animal welfare (EU Convention on the protection of animals revised directive 86/609/EEC) for reasons unrelated to this study, were potential candidates. With the owner's consent, they were screened for any form of elbow pathology, based on orthogonal radiographs, and subsequently frozen at −20°C, if they showed no abnormal finding. Prior to further processing, the cadavers were thawed for 48 h at room temperature. For each specimen, the determination of whether the left or right elbow joint would be assigned to the arthroscopy group (group 1) or the arthrocentesis group (group 2) was made through a random assignment process using a coin toss. Regardless of the group assignment, both elbow joints were clipped as for a standard arthroscopic procedure. For elbow arthroscopy, the dogs were positioned in lateral recumbency, with the elbow joint to be operated on at the edge of the table. Elbow arthroscopy was performed using the standard medial portals and a 1.9-mm needle arthroscope and a 2.4-mm arthroscopic sleeve (NanoScope, Arthrex Inc., Naples, FL 34108, USA). 23 To achieve a permanent flow of fluid, a 1-L Ringer infusion solution (Ringer-Infusionslösung B. Braun, B. Braun Melsungen AG, 34209 Melsungen, Germany) was connected to the arthroscopic sleeve using an infusion set (Infusionsset, Eickemeyer-Medizintechnik für Tierärzte KG, 78532 Tuttlingen, Germany) and a pressure cuff (Metpak, Rudolf Riester GmbH, 72417 Jungingen, Germany). A 20-gauge hypodermic needle was placed into the olecranon fossa as an egress cannula (Hypodermic Needle; Covetrus BV, 5431 SLCuijk, Netherlands). The pressure of the system was kept constant between 40 and 50 mmHg during the arthroscopy. 5 18 23 After intraarticular placement of the camera, the instrument portal was established under visual control, just caudal to the medial collateral ligament. After gentle enlargement of the working portal with a curved mosquito forceps, an arthroscopic palpation hook was inserted and routine exploration of the joint was performed, including palpation of the medial joint structures. All arthroscopic procedures, including arthrocentesis and portal placing, were performed by a board-certified surgeon (P.S.). 1 At the end of the diagnostic arthroscopy and following removal of the scope, the arthroscopic sleeve, and the palpation hook, aspiration of the remaining irrigation fluid from within the joint was performed via the egress cannula placed into the olecranon fossa. Subsequently, 2.5 mL of undiluted contrast media (Xenetix ® 300; 300 mg Iod/mL, Guerbet, Roissy CdG Cedex, France) were injected via the same needle using a 3-mL syringe (Omnifix® Luer Solo 3 mL, REF 4616025V, B. Braun Melsungen AG, 34209 Melsungen, Germany), ensuring true intraarticular contrast media application. After this, the dogs were turned onto the opposite side and arthrocentesis at exactly the corresponding location as the para anconeal egress cannula on the arthroscopy side was performed. Intraarticular needle placement was confirmed by aspiration of synovial fluid and 2.5 mL of contrast media were injected into the joint. Without further delay, axial CT imaging (SOMATOM go.now, Siemens, Erlangen, Germany) of both elbow joints was acquired (KVP 130 V; mA 160; 0.6 mm slice thickness; 0.30 mm increment). To allow for standardized imaging of all investigated elbow joints, the cadavers were positioned in dorsal recumbency, with both front limbs in parallel and the elbow joints at 135 degrees, using a custom-made positioning device ( Fig. 1 ). Time between injection of contrast media after arthroscopy as well as arthrocentesis and the start of CT scanning was recorded for every specimen.

**Fig. 2:**
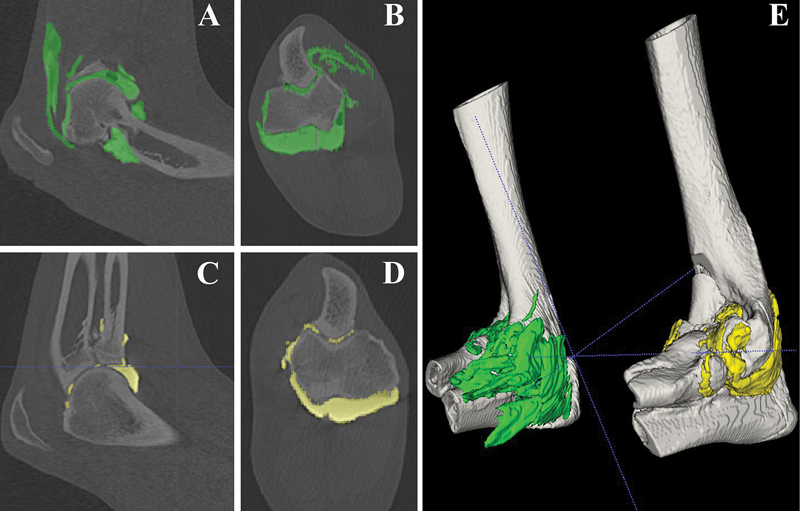
Computer tomographic (CT) measurements of intra- and periarticular contrast fluid. ( **A** ) Sagittal and ( **B** ) coronal views of a CT scan of an elbow joint after contrast fluid injection and elbow arthroscopy with the intra- and periarticular contrast media–enhanced fluid marked in green. ( **C** ) Sagittal and ( **D** ) coronal views of a CT scan of an elbow joint after contrast fluid injection without elbow arthroscopy with the intra- and periarticular contrast media–enhanced fluid marked in yellow. ( **E** ) Three-dimensional model of the elbows displayed in **A** , **B** , **C** , and **D** .

To allow monitoring of contrast medium leakage over time, consecutive scans were performed at seven predefined time points: T0, T1 (T0 + 2 minutes), T2 (T0 + 4 minutes), T3 (T0 + 6 minutes), T4 (T0 + 8 minutes), T5 (T0 + 10 minutes), and T6 (T0 + 15 minutes). These time intervals were chosen to investigate whether a possible extravasation occurs exclusively just after the operation or also in the further short-term course. To detect a possible loss of contrast medium from the joint, both the total volume and the total intensity of the contrast medium-enhanced fluid were measured both intra- and extraarticularly. For each elbow scan, one single investigator (L.R.) calculated 3D renderings of the intraarticular and extracapsular contrast media–enhanced fluid, using the open-source software ITK-SNAP (Version 3.8.0, July 22, 2022). 24 25 26 This was performed in a nonrandomized and nonblinded manner. In each cross-sectional image, the intra- and extraarticular contrast media–enhanced fluid was manually segmented using the software's internal brush tool. The visual difference in image contrast between soft tissues, the contrast media–enhanced fluid, and bone structures was used to delineate the region of interest. Finally, the volume (mm ^3^ ) of the segmented intra- and extraarticular contrast media–enhanced fluid, as well as its mean intensity (Hounsfield Units, HU), was calculated from the voxel volumes segmented in the outlined manner. This process was performed for each elbow, in each specimen, and at all seven time points. The volumes segmented in this way represent the sum of intraarticular and extraarticular contrast medium–enhanced fluid. In the same way, the segmented intensity represents the intensity for the entire measured volume, including intraarticular and extraarticular contrast-enhanced fluid. To simplify the phrasing in the following, the terms volume and intensity of the contrast medium–enhanced fluid represent the sum of intraarticular and extraarticular volume and the corresponding intensity. The two segmented contrast media–enhanced fluid volumes of each specimen at a certain time point were labeled accordingly as arthroscopy (AS) in green, or arthrocentesis (AC) in yellow ( Fig. 2 ).

### Data Analysis

The effect of the arthroscopy treatment on fluid volume and fluid intensity was first tested using two separate linear models, with time as a covariate. For each model, we tested for the significance of the treatment variable using likelihood ratio tests, which consisted in comparing the goodness of fit of models fitted with or without the variable. Linear mixed effect models were then performed to further test for potential basal differences among dogs in fluid volume and intensity, with arthroscopy treatments and time as fixed effects and the specimen ID as a random effect. Large random effect variance would indicate large variance among dogs in their basal fluid intensity or volume. *P* -values lower than 0.05 indicated statistically significant results. Statistical analyses were performed using R and the lme4 package. 27 Response variables were standardized prior to analyses.

## Results

A total of eight dogs, meeting the inclusion criteria, were included in the study. The median age of the dog was 10.2 years (5.6 to 16.8 years) and the median weight was 35.0 kg (26.0 to 42.0 kg). There were two females, two males, three neutered females, and one neutered male. Breeds are shown in Supplementary Table S1 (available in the online version only). As a result of randomization, arthroscopy was performed on six right and two left elbow joints. The average time interval between the intraarticular application of the contrast agent and the start of the CT scans (T0) was 10 minutes (SD 2).

The volume of contrast media–enhanced fluid measured over all seven time points was considerably larger in the arthroscopy group compared with the arthrocentesis group (likelihood ratio test: volume χ ^2^ = 144.6, df = 1, *p* -value < 0.0001) with an average of 11,663 mm ^3^ (S.D. 221.13) of fluid in the arthroscopy group and 6,548 mm ^3^ (S.D. 108.23) in the arthrocentesis group. On average volume was greater by 5,115 mm ^3^ in the arthroscopy group (linear model standardized estimate: 1.69, std. error 0.10) ( Table 1 and Supplementary Fig. S1 , available in the online version only).

**Table 1:** Mean measured volumes and intensities of contrast media enhanced–fluid in arthroscopy and control group over the seven time points

	T0		T1 (= 2 minutes)		T2 (= 4 minutes)		T3 (= 6 minutes)		T4 (= 8 minutes)		T5 (= 10 minutes)		T6 (= 15 minutes)	
	Vol.	Int.	Vol.	Int.	Vol.	Int.	Vol.	Int.	Vol.	Int.	Vol.	Int.	Vol.	Int.
**Arthrocentesis**	6,441.73	2,116.41	6,533.3	2,076.38	6,466.65	2,099.89	6,560.09	2,074.94	6,550.87	2,077.36	6,545.45	2,044.89	6,738.45	1,997.35
**Arthroscopy**	11,347.25	786.05	11,411.19	767.4	11,552.97	746.29	11,659.41	736.15	11,549.15	740.13	11,788.24	714.17	12,335.31	685.12

Abbreviations: Int., intensity in Hounsfield Units; min., minutes; T, time point (0 to 6) in minutes; Vol., volume in mm ^3^ .

The intensity of the contrast media–enhanced fluid measured across all seven time points was significantly lower in the arthroscopy group (mean: 739.33 HU, SD: 246.68) compared with the arthrocentesis group (mean: 2069.60 HU, SD: 605.34), as indicated by a likelihood ratio test (χ ^2^ = 135.9, df = 1, *p* < 0.0001). On average, the measured intensity of contrast media–enhanced fluid was lower by 1,330 HU in the arthroscopy group (linear model standardized estimate: −1.66, std. error 0.11) ( Table 1 and Supplementary Fig. S2 , available in the online version only).

**Fig. 3:**
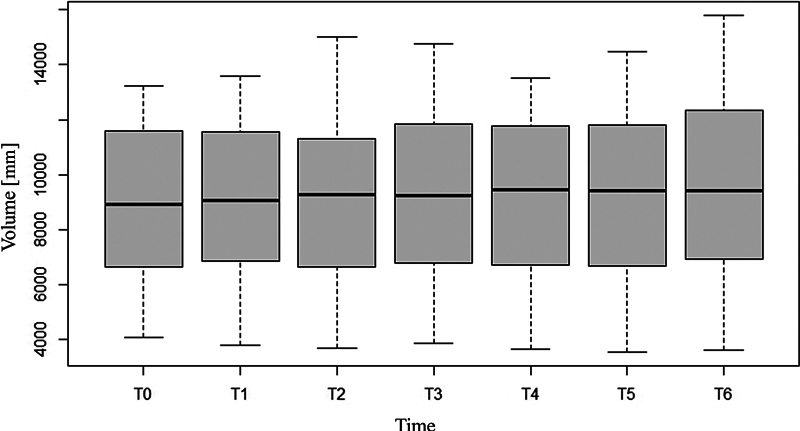
Contrast-enhanced fluid volume response for all dogs over time: On the y-axis the volume in mm ^3^ is shown. The seven time points (T0 to T6) are shown on the x-axis. The boxplots in this graph show no significant alteration of volume over time.

**Fig. 4:**
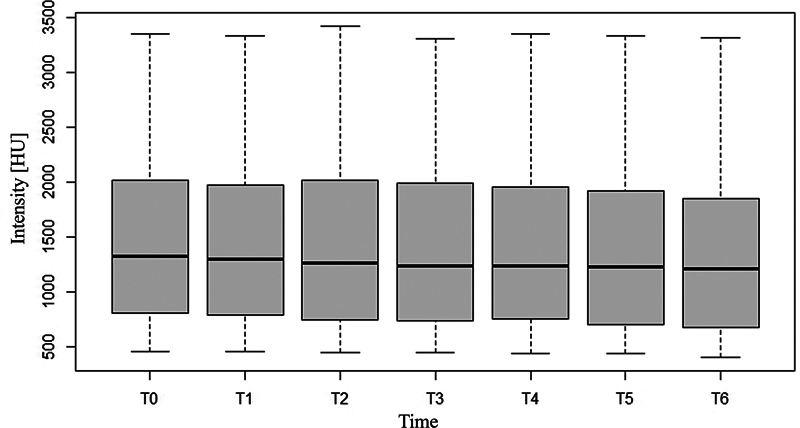
Contrast-enhanced fluid intensity response for all dogs over time: On the y-axis the intensity in Hounsfield Units (HU) is shown. The seven time points (T0 to T6) are shown on the x-axis. The boxplots in this graph show no significant alteration of intensity over time.

There was no significant change in the measured volume or intensity of contrast media–enhanced fluid over the study period of 15 minutes, for either the arthroscopy or the arthrocentesis group (likelihood ratio test: volume χ ^2^ = 1.69, df = 6, *p* -value = 0.94, intensity χ ^2^ = 0.63, df = 6, *p* -value = 0.99) ( Table 1 , Figs. 3 and 4 ).

Linear mixed effect models revealed high variance among dogs and high consistency within each dog regarding how they responded to the treatment, in both contrast media–enhanced fluid volume (random intercept variance effect: 0.1044, corresponding to a 0.35 intraclass correlation) and intensity (random intercept variance effect: 0.2457, corresponding to a 0.74 intraclass correlation) ( Supplementary Fig. S1 and S2 , available in the online version only). Mixed effect model estimates for the effect of the arthroscopy treatment on fluid volume and intensity are remarkably close to estimates of simpler linear models, with smaller estimated standard deviations in mixed effect models (volume: mixed model standardized estimate: 1.69, std. error 0.08; intensity: mixed model standardized estimate: −1.67, std. error 0.05), arguing for a strong effect of arthroscopy on fluid volume and intensity in linear mixed effect models.

## Discussion

In our study setup, a significant extravasation of contrast medium was observed in the arthroscopy group, which supports the first hypothesis. However, the second hypothesis was not confirmed, as neither the intensity of the contrast medium–enhanced fluid nor the volume changed within the first 15 minutes following application.

In the control group, the contrast agent was clearly delineated from the surrounding periarticular tissue in the CT images and showed a high intensity in all cases. It can be assumed that the injected contrast agent remains condensed inside the capsular space leaving the joint capsule intact. If, on the other hand, the distribution pattern of the contrast agent in the arthroscopy group is compared with the control group, no clear delineation and a diffuse spread in the periarticular tissue was observed. As a result, the measured fluid volume increases and the measured fluid intensity decreases.

Since, as in the study of Blumhagen and colleagues, applied irrigation fluid was removed after the arthroscopy and the contrast medium was only administered afterwards, it can be assumed that the increase in volume and the decrease in intensity are caused by the contrast medium leaking out of the intraarticular space and spreading into the periarticular tissue. 18 Extravasation of irrigation fluid is a well-described phenomenon during and after arthroscopy in both humans and dogs. 19 20 21 In human orthopedic traumatology, the so-called saline load test is used to visualize even small traumatic joint capsule injuries. 28 29 30 A defined amount of sterile saline solution is injected into the affected joint to show leakage of this fluid, which would indicate a lesion of the joint capsule. This procedure has been shown to have a high sensitivity and a high specificity. 28 29 30 The extravasation of fluids during saline load test and during irrigation in arthroscopic procedures through lesions of the joint capsule support our hypothesis that a leakage of fluids through the arthroscopic ports occurs.

The decrease in the intensity of the contrast medium in the arthroscopy group can be explained in a similar way. Although the contrast fluid in the control group remains condensed in the joint space and therefore no dilution occurs, in the arthroscopy group, there is a loss of intensity due to extravasation and associated increase in volume due to a higher distribution between the surrounding soft tissues. In addition, a certain dilutional effect on the injected contrast agent, which decreased the intensity in the arthroscopy group, cannot be excluded. Both the intraarticular synovium and the irrigation fluid applied during arthroscopy may cause such a dilutional effect. In both human and veterinary studies, synovial and effusion fluid was removed by puncture prior to injection of contrast medium for CT arthrography. 31 32 33 Despite this procedure, Samii and colleagues 33 noted that it was probably not possible to remove all of the fluid and that this could have led to a potential dilutional effect of the contrast medium, resulting in poorer contrast medium resolution. However, since a dilutional effect by synovia should also influence the intensity in the control group to the same extent, no influence on the results of the study should be expected. After the arthroscopic procedure, care was taken to evacuate the joint via arthrocentesis under visual control to the best possible extent. Investigating the possible dilutional effect of irrigation fluids after arthroscopy, Stopka and colleagues evaluated intraarticular injected trypan blue solution in a human knee cadaveric model. 34 After a routine arthroscopic exploration, an attempt was made to remove all irrigation fluid from the joint via the empty arthroscopy cannula by moving the joint and massaging the fluid out before the solution was injected. After several range of motions, samples of the intraarticular fluid were obtained and spectometrically analyzed and revealed a dilutional effect of approximately 27%. 34 Considering the results of our study with a percentage decrease in intensity by 64.3%, the demonstrated dilutional effect is approximately twice as high as described by Stopka and colleagues, confirming the hypothesis of dilution due to extravasation.

It is difficult to say why in our experiment the dilutional effect is almost twice as large, especially since we also tried to remove the irrigation fluid before injection. Possible explanations are that the removal of irrigation fluid was not effective enough in our study and that the measurement methods are different as in the mentioned study only the intraarticular fluid concentration was compared with the original solution. In our case, however, all contrast fluid, including the extracapsular component, was also measured, so that not only a dilutional effect due to remaining irrigation fluid, but also a dilutional effect due to distribution into the surrounding tissue was taken into account.

With our manual measurement of the intra- and extracapsular contrast agent, we observed that in some areas there is an overlap in the intensities measured in HU between the different tissues and the diluting contrast fluid. When trying to automatically calculate the contrast agent volumes based on defined thresholds of HU values, there were frequent errors compared with the manual measurements in our work. Unintentional additions as well as subtractions of areas were observed in the automated procedure compared with the manual method. This observation in our study could explain the different results compared with the work of Blumhagen and colleagues which used an automated procedure. 18

Various therapeutic agents such as local anesthetics, or orthobiologics, e.g., platelet-rich plasma, have been described for intraarticular application. Several studies describing a positive effect of local anesthetics injected intraarticularly after arthroscopy. 6 7 8 One possible explanation is that local anesthetics can still achieve an adequate effect after extravasation due to resorption in the surrounding tissues. This might be different for specific agents in the treatment of osteoarthritis. The use of orthobiologics for the therapy of cartilage lesions or osteoarthritis shows good medium- to long-term effects in dogs. 10 13 16 35 36 As far as the mechanism of action of platelet-rich plasma is understood, it locally stimulates a variety of processes that promote tissue healing, such as cell recruitment and angiogenesis, via various growth factors. 37 38 39 Due to these effects in the area of the affected lesion, local application at an appropriate concentration appears to be indicated even if, according to the current literature, no clear statements can be made about specific concentrations. 37 Taking these considerations into account and given the dilution effect of the contrast medium–enhanced fluid volume of 64.3%, the question must be asked whether enough of the therapeutic fluid remains at the desired location and if an effective concentration can be achieved with an injection immediately after elbow arthroscopy. In a recent study by Scharpf and Theyse, no significant clinical effect was observed following intraarticular injection of autologous conditioned plasma (ACP) after elbow arthroscopy and subtotal coronoidectomy compared with a control group with injection of a placebo. 40 Future studies should evaluate which concentrations of platelet-rich plasma are needed intraarticularly to achieve an adequate effect level. Until then injection of orthobiologics might be considered at a later time point.

The fact that neither intensity nor volume changed significantly over the course of the various measurement points indicates that the main effect of extravasation already occurred directly at time point T0. The causative extravasation therefore occurred directly after the arthroscopy and injection of contrast media. One possible explanation as to why even more fluid did not escape from the joints over the further course of time could be a lack of movement. In an in vivo scenario, there would most likely be even more extravasation due to the movement of the joints.

Our results also revealed high variance among dogs in each treatment group but high consistency in how each dog responded to the treatment. This means that dogs which showed higher fluid volume or intensity in the control group also showed higher fluid volume and intensity in the arthroscopy group. We speculate that the physiology of the dogs has an influence on the measured volumes and intensities but does not influence the ratio of extravasation.

Several limitations must be considered when interpreting the results of this study. First, the ex-vivo character of this study does not allow direct translation to the clinical field. The use of elbow joints of previously frozen cadaveric dogs might have compromised the integrity of the joint capsule. However, as the same dogs were used as their control, expected compromises to the joint capsule should occur to the same amount in the control group. As the use of thawed joints showed no adverse effects in the work of Samii and colleagues and Gendler and colleagues with an similar setup, we estimate the risk for effects in our study to be low. 33 41 As previously discussed joint movement after injection could have a significant effect on extravasation and should therefore be considered in future studies. 18 34 Since the contrast medium was administered without monitoring the application pressure and without blinding, this aspect could also represent a limitation. As the injection was easy and without resistance in all cases, an effect on increased extravasation due to increased application pressures appears to be low. But a certain variance in the application pressures during manual administration cannot be ruled out. Also, the widening of the arthroscopic ports with mosquito forceps may have contributed to an increased risk of extravasation. But as this is common practice in many institutions and is a described technique in the literature, we wanted to include this effect in our study setup. 18 23 How great the effect of the widening really contributes to leakage cannot be clearly stated at this point. Future studies should evaluate whether there is a difference between widening and non-widening of arthroscopic portals. In addition, the pressure of 40 to 50 mmHg used for irrigation during arthroscopy can be criticized as relatively high. These pressures are described in the literature and are commonly used in practice, and we therefore wanted to consider the possible effects of these pressures in our experimental setup. 5 18 23 Whether there is a possible influence of different irrigation pressures on the extravasation behavior is beyond the scope of this study. Lastly, all CT measurements were performed by a single, non-blinded investigator, which might have caused a certain degree of bias.

## Conclusion

In conclusion, we were able to show a significant extravasation of intraarticularly injected contrast fluid after arthroscopy by means of an increased volume and a decreased intensity in this cadaveric model. No significant effect of time on the contrast fluid volume and intensity could be shown. Injection of liquid therapeutics, e.g., orthobiologics, at a later time point after arthroscopy should be considered.
