# Supplementary material for: Extravasation of Intraarticular Fluid Injection Following Canine Elbow Arthroscopy: A Cadaveric Study
Source: Vet Comp Orthop Traumatol. 2025 Jul 16;39(1):29–37. doi: 10.1055/a-2648-6856 (PMC12811057; doi:10.1055/a-2648-6856)
Supplement: Supplementary file 1 — Supplementary Material [file 10-1055-a-2648-6856-s24100092.pdf]

Supplementary Fig. S1 Contrast-enhanced fluid volume response in the arthroscopy and control groups. On the y-axis the volume in mm^3 is shown. The control (in yellow) and the arthroscopy (in green) groups are shown on the x-axis. The volume values of each individual are also shown as dots. The corresponding elbow joints of an individual in both groups are connected with lines for improved visualization. The boxplots in this graph show a significantly increased volume of contrast media–enhanced fluid in the arthroscopy group compared with the control group.

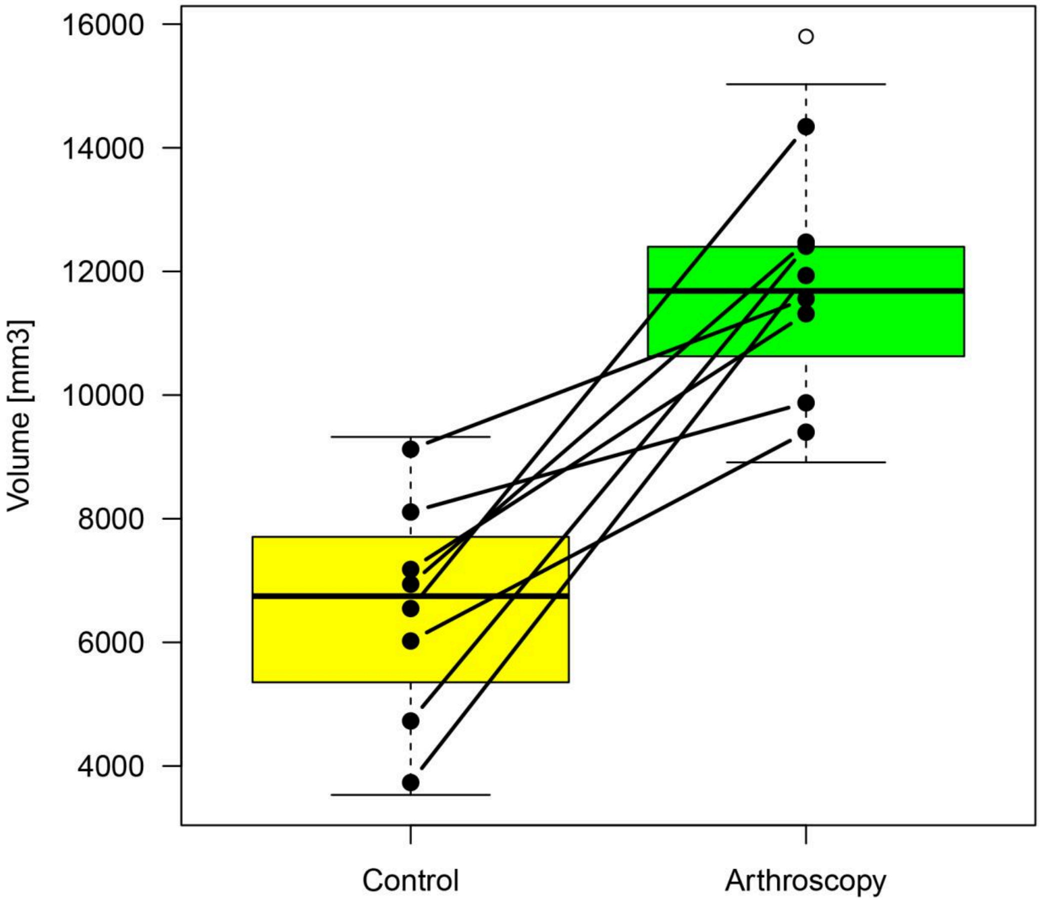

Supplementary Fig. S2 Contrast-enhanced fluid intensity response in the arthroscopy and control groups. On the y-axis the intensity in Hounsfield Units (HU) is shown. The control (in yellow) and the arthroscopy (in green) groups are shown on the x-axis. The intensity values of each individual are also shown as dots. The corresponding elbow joints of an individual in both groups are connected with lines for improved visualization. The boxplots in this graph show a significantly decreased intensity of contrast media–enhanced fluid in the arthroscopy group compared with the control group.

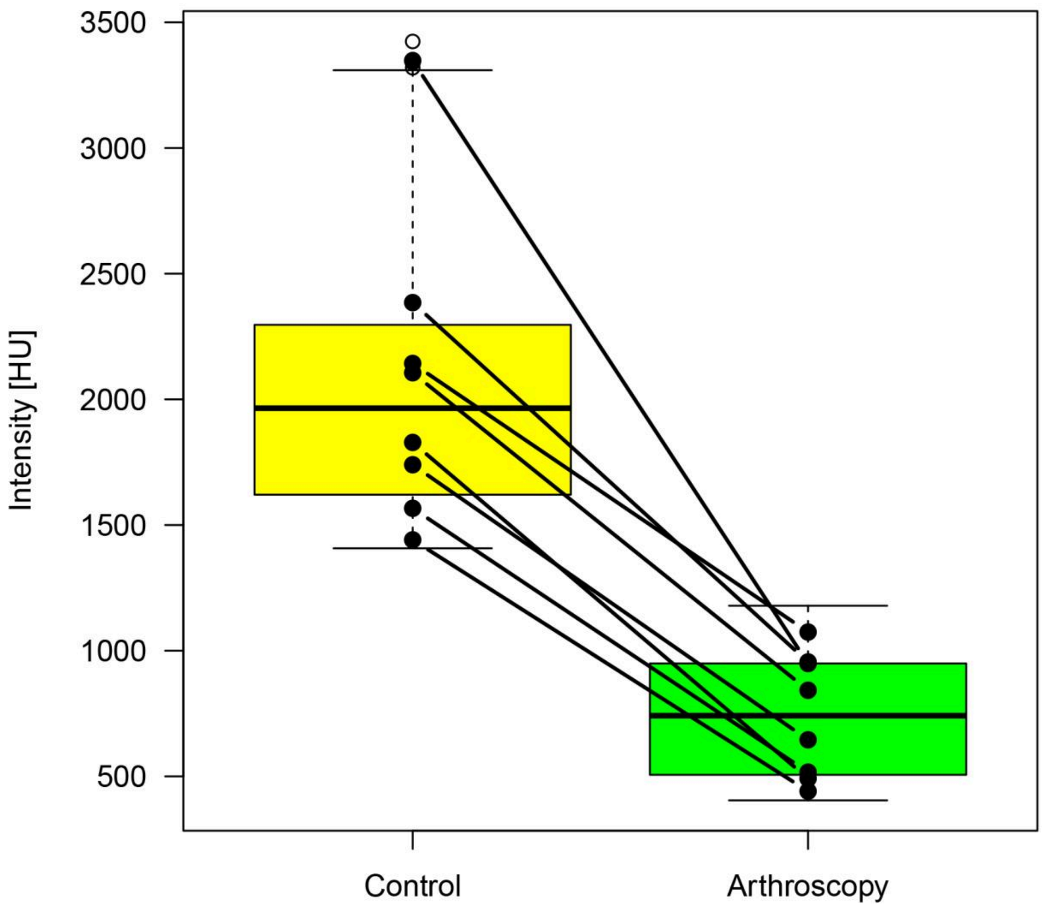

Supplementary Table S1 Specifications of the dog specimens used

Study Nr.:	Age: [y]	Breed:	Weight: [kg]	Sex:	Reason of death/Euthanasia
1	7.8	Dobermann	30	female	Acute Cardial Arrest
2	11.7	Mixed Breads	26	male	Pituitary Neoplasia
3	13.2	Mixed Breads	45	female, spayed	Status Epilepticus
4	9.8	Mixed Breads	37	male, spayed	Hemangiosarcoma
5	9.0	Mixed Breads	42	female, spayed	Neoplasia Cranium
6	16.8	Dalmatine	27	female, spayed	MODS, Neoplasia
7	5.6	Swiss Mountaindog	41	male	Satus Epilepticus
8	7.9	Mixed Breads	32	female	Bleeding Spleen

Abbreviations: kg, kilogram; MODS, multiorgan dysfunction syndrome; Nr., number; y, years.
